# Large retroperitoneal cystic lymphangioma mimicking mesenteric cyst: a case report

**DOI:** 10.11604/pamj.2022.42.115.30777

**Published:** 2022-06-10

**Authors:** Deepak Rajput, Kandhala Srikanth, Amit Gupta, Arvind Kumar, Sanketh Edem, Lena Elizabath David, Krishna Sai Bhukya

**Affiliations:** 1 Department of General Surgery, All India Institute of Medical Sciences Rishikesh, Dehradun, Uttarakhand, India,; 2 Department of Pathology, All India Institute of Medical Sciences Rishikesh, Dehradun, India

**Keywords:** Retroperitoneal cyst, cystic lymphangioma, primary retroperitoneal mass, case report

## Abstract

Cystic lymphangioma is a benign tumour that occurs secondary to obstruction of lymphatic channels. Its appearance in the paediatric age group is quite common, but adulthood presentation is infrequent. Common locations are head and neck areas, whereas intra-abdominal occurrence is rare. To date, a few retroperitoneal cystic lymphangioma cases have been reported. A pre-operative clinical detection is always confusing, and most often, the diagnosis rests over the intraoperative findings and histopathological examination. The cyst's complete surgical resection remains the treatment of choice in patients with bulky, rapidly growing lesions or symptoms. Herein, we report a large retroperitoneal cystic lymphangioma that mimicked the mesenteric cyst clinically.

## Introduction

According to the image findings, primary retroperitoneal masses do not originate from a specific retroperitoneal organ and are divided into two major groups: solid and cystic. Retroperitoneal cysts are rare in incidence and can be neoplastic or non-neoplastic. Neoplastic retroperitoneal lesions include lymphangioma, mature teratoma, cystic mesothelioma, cystadenoma, and cystadenocarcinoma. On the contrary, epidermoid cyst, nonpancreatic pseudocyst, bronchogenic cyst, lymphocele, haematoma, and urinoma are non-neoplastic lesions [1].

The incidence of retroperitoneal lymphangioma is about 1%. Clinical presentation is usually asymptomatic because of its slow growth rate. Symptomatic cases, although few, present with abdominal pain, lump abdomen, and local compressive symptoms. Large cysts may present as acute abdomen with features of intestinal obstruction, torsion, or bleeding [2]. Because of its rarity and non-specific presentation, diagnosis alone by clinical examination and radiological imaging is often difficult. It usually requires either open or laparoscopic complete excision and histopathological examination for confirmatory diagnosis.

## Patient and observation

**Patient information:** a 65-year-old male patient of Indian ethnicity presented in the general surgery outpatient department with a left upper abdominal lump associated with dull aching continuous non-radiating pain around the umbilicus over three months. On questioning, he denied any history of vomiting, haematemesis, constipation, bleeding per rectum, melena, fever, anorexia, loss of weight, or difficult micturition.

**Clinical findings:** physical examination of the patient revealed a moderate built and an apparent fullness over the left hypochondrium that extended towards the umbilicus. An ill-defined lump of 10 x 7 cm, spanning the entire left hypochondrium and extension towards the umbilicus and the epigastrium, could be palpated on abdominal examination. The swelling was non-tender, smooth-surfaced, firm in consistency, moving horizontally but immobile vertically with no local temperature rise. On percussion, a zone of resonance was noteworthy around the lump. Examination of the bilateral renal angles, lumbar regions, and back was unremarkable. No generalized lymphadenopathy was present.

**Figure 1:**
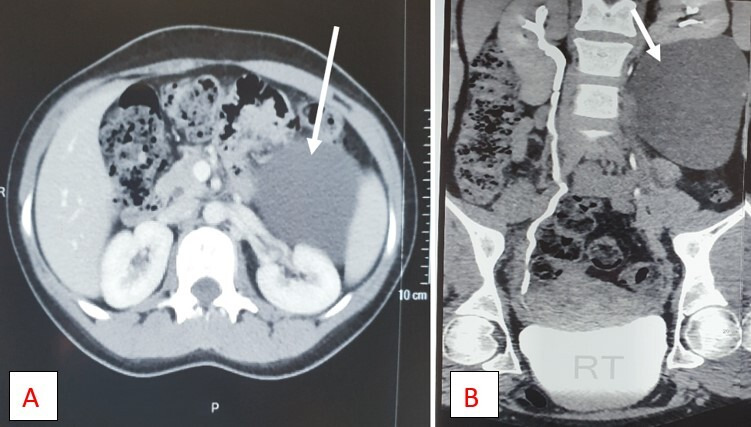
computed tomography scan showing: A) cystic lesion in left anterior pararenal space (arrow) in axial view; B) the lesion displacing the proximal and the midpart of the left ureter medially in coronal section

**Diagnostic assessment:** considering mesenteric cyst and retroperitoneal cyst as differential diagnoses, an ultrasound abdomen was done that showed a cystic mass of size 12 x 11 cm with septations noted on the left side above the level of the umbilicus. Spleen, pancreas, and bilateral kidneys are normal. Contrast-enhanced computed tomography (CECT) showed evidence of a well-defined thin-walled lesion of 13x10x12 cm in the left anterior para renal space. The lesion abutted the left kidney and renal vessels posteriorly, the spleen laterally, the left lateral abdominal wall anteriorly, and the bowel loops medially. The adjoining mass had also displaced the left ureter medially in its upper and mid-part without any evidence of hydronephrosis and psoas muscle involvement (Figure 1).

**Diagnosis:** retroperitoneal cyst.

**Figure 2:**
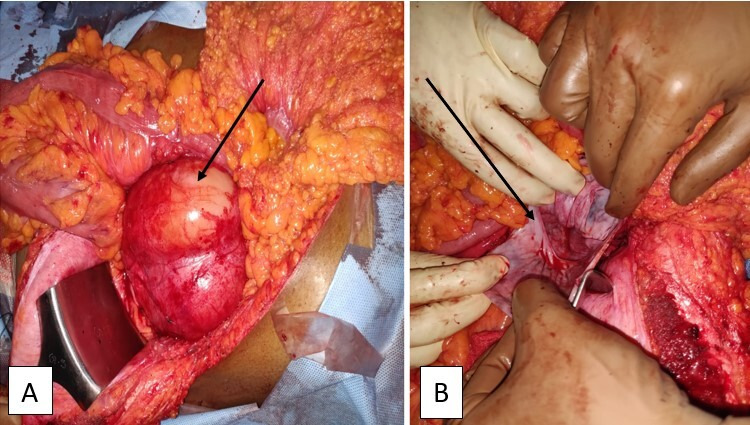
intraoperative photograph revealing: A) a large infracolic bilobed cyst covered by mesentery anteriorly; B) the thin glistening posterior adherent cyst wall after dissection and partial excision

**Figure 3:**
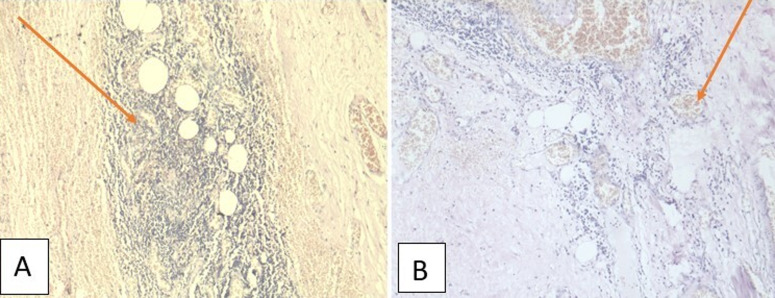
microphotograph after Haematoxylin and Eosin stain demonstrating: A) lymphoid aggregates in cyst wall at 400X magnification; B) the lymphatic channels in fibrous meshwork at 100X view

**Therapeutic intervention:** the patient underwent an exploratory laparotomy through a left subcostal incision with a lower midline extension. Intraoperatively, a thin-walled cystic lesion of 10 x 7 cm from the retroperitoneum was noted below the left kidney level (Figure 2). The left ureter was pushed medially and embedded in the cyst wall. On reflecting the left colon medially, the displaced superior mesenteric vein was adherent to the anterior cyst wall. Spleen, bowel, and mesentery were normal. Around 400 ml of thick and cloudy fluid was aspirated from the cyst to facilitate manipulation. The sample sent for the cytological examination came negative for malignant cells. During the dissection, the origin of the cyst was densely adherent to the abdominal aorta over the left posterolateral side. Hence, a partial excision with eversion and suturing of the remaining wall was undertaken. The excised cyst wall sent for a histopathological examination showed the glistening, pale-coloured cyst wall with single separation on gross analysis. Finally, the microscopy demonstrated a fibrous cyst wall with variable-sized lymphatic channels and focal lymphoid aggregates that led to the final diagnosis of cystic lymphangioma (Figure 3).

**Follow-up and outcomes:** the patient´s postoperative course was uneventful, and he could go home on postoperative day 4 with a healthy wound. There were no abdominal complaints or any wound complications at the one-month follow-up.

## Discussion

Lymphangioma is a rare type of benign malformation of lymphatic channels. Its incidence is expected in the paediatric population but rare in the adult population. For the first time, cystic lymphangioma was reported in 1913 by *GAUDIER and GORSE*. It does not show any sex preference, with 90% of cases being in their first two years of life. Common locations of occurrence are head and neck (75%) and axilla (20%). Approximately 5% are intra-abdominal, where it can arise from mesentery, retroperitoneum, and greater omentum [2]. Despite the many theories proposed on its development, their frequent occurrence within the primitive lymph sacs containing stagnant lymph strongly supports the origin from the sequestered lymphatic tissue that had failed to establish communications with normal lymphatic channels and the venous system [3]. Based on the size of the lymphatic spaces, these lymphangiomas fall into either cavernous, capillary, or cystic types.

Clinical presentation is asymptomatic in most cases. When symptomatic, they present as vague abdominal pain, slowly growing mass, nausea/vomiting, and anorexia [4]. Large cysts may present with intestinal/urinary obstruction or adjacent viscera dislocation. Unusual complications include torsion, rupture, bleeding, and ascites. The clinical diagnosis of cystic lymphangioma originating from the retroperitoneum is often very challenging, and the differential diagnoses include cystic lesions arising from the spleen, kidney, pancreas, and ovaries [5]. Some cysts, particularly those from the deep retroperitoneal space and long narrow stalks, are often confused with mesenteric cysts and present with the classical Tillaux triad - soft fluctuant swelling in the umbilical region, zone of resonance all around the swelling, and vertical movement in relation to the mesentery. The described patient had all the above features.

Radiological investigations like ultrasound, computed tomography (CT), and magnetic resonance imaging (MRI) complement. An ultrasound shows a sharply delineated fluid-filled cystic lesion with septations. CT and MRI can demonstrate uni or multiseptated lesions, relationships with the adjacent organs, and characteristic water density fluid content [6]. In the case discussed herein, CT revealed a well-defined left suprarenal cystic lesion of 13.7 x 13.5 x 13.8 cm with enhancing septum and few focal mural calcifications. An image-guided biopsy is not advisable due to the risk of intra-abdominal spillage if an undiagnosed malignancy exists.

Complete excision of the cyst is the standard gold treatment [7]. Other management options include aspiration, cyst-enterostomy, and marsupialization, which are obsolete due to the risk of recurrence. Involvement or origin of the cyst from any intra-abdominal organ warrants partial excision but has a chance of recurrence [8]. We, too, could only do a partial surgical resection as the cyst wall was densely adherent to major vessels and left ureter in the retroperitoneum.

The final tissue diagnosis is arrived at after histopathological examination of the excised cyst. The characteristic features include an endothelial lined well-circumscribed cyst wall containing focal lymphoid tissue aggregates and stroma composed of collagen fibrous meshwork [9]. Accordingly, in our case, the microscopic examination showed a fibrous cyst wall with variable-sized lymphatic channels and focal lymphoid aggregates. Henceforth, a diagnosis of cystic lymphangioma was made based on the intraoperative and microscopic findings.

**Patient perspective:** the patient shared his perspective on the treatment in terms of pain relief and disappearance of the swelling.

**Informed consent:** the patient gave written informed consent to publish his anonymized information in this article.

## Conclusion

Cystic Lymphangioma is a rare benign tumour that occurs due to abnormal lymphatic channels. Adult and intra-abdominal incidence of cystic lymphangioma is infrequent. Because of rarity and non-specific clinical presentation, it poses diagnostic challenges. It often requires surgical excision and histopathological examination for a confirmed diagnosis.
